# O.2.3-6 Free-living patterns of physical activity and sedentary behaviour of workers from the manufacturing industry of micro, small and medium enterprises: the Happy-OPA project

**DOI:** 10.1093/eurpub/ckad133.132

**Published:** 2023-09-11

**Authors:** Anna Codina-Nadal, Anna Puig-Ribera, Antoni Corominas, James Mckenna

**Affiliations:** University of VIC, Spain; University of VIC, Spain; University of VIC, Spain; Leeds Beckett University; anna.codina@uvic.cat

## Abstract

**Background:**

Occupational physical activity (OPA) may not report the same health benefits as leisure time physical activity (LTPA). While LTPA is beneficial for all workers, OPA can be detrimental for health. Most importantly, LTPA might have different health effects for different occupational groups (low versus high OPA jobs). It is also unclear the influence LTPA or leisure time sedentary behaviour (LTSB) might have on the health, wellbeing and productivity of workers with high OPA jobs. This project will (i) study the free-living patterns of (occupational and leisure time) physical activity (PA) and sedentary behaviour (SB) in workers from the manufacturing industry. Then, we will (ii) investigate associations between free-living patterns of PA and SB with mental health, happiness, absenteeism and work-related injuries of manufacturer workers.

**Methods:**

Cross-sectional study. Employers and occupational risk prevention services will randomly invite workers (aged 18 to 65) from manufacturing industries of micro, small and medium enterprises in Central Catalonia to participate (n = 220). Participants will wear an ActivPAL device for 7 days and will complete a questionnaire on general health, life balance between work and family life, mental well-being, mental health, work-related happiness and absenteeism.

**Variables:**

(1) physical activity pattern (activPAL3TM), (2) demographic data and lifestyles (food, tobacco and alcohol habits (ESCA 2022), (3) impact of work on employees' health, absence for illness (5th and 6th European Survey of Working Conditions), (4) well-being (Health Questionnaire, SF-36), (5) Work Health Balance (WHB Questionnaire), (6) Mental Health (Patient Health Questionnaire, PHQ-9), (7) Happiness (Pemberton Happiness Index). The group will be analysed on the average value of the variables.

**Discussion:**

Identifying the free-living patterns of physical activity and sedentary behaviour of workers with high OPA and its associations with health, will provide preliminary data to develop tailored PA (occupational and non-occupational) interventions to this occupational group.

**Support / Funding Source:**

Grants for the recruitment of research staff (Agency for the Management of University and Research Grants, co-financed by the European Union).

